# FuDiCo: Gene Fusion-Initiated Path Propagation for Disease Comorbidity Prediction

**DOI:** 10.3390/cimb48060622

**Published:** 2026-06-16

**Authors:** Ashwag Altayyar, Li Liao

**Affiliations:** Department of Computer and Information Sciences, University of Delaware, Newark, DE 19716, USA; ashwag@udel.edu

**Keywords:** disease comorbidity prediction, gene fusion, protein–protein interaction network, subgraph representation learning, influence propagation, graph analysis

## Abstract

Disease comorbidity—the co-occurrence of two or more diseases in the same individual—has gained growing attention due to its association with adverse clinical outcomes and increased treatment complexity. Recent subgraph-based approaches for disease comorbidity prediction model disease modules as subgraphs induced by disease-associated genes in the protein–protein interaction (PPI) network and learn disease representations from subgraph topology. However, these approaches are constrained by incomplete disease–gene annotations, which may obscure important molecular relationships between diseases. Accordingly, disease comorbidity may also be influenced by molecular events beyond annotated disease genes, such as gene fusion events that have emerged as important contributors to disease mechanisms. Motivated by the role of gene fusions in disease development, we introduce Gene Fusion-Initiated Path Propagation for Disease Comorbidity Prediction (FuDiCo), a framework that models comorbidity through influence propagation over the PPI network. FuDiCo represents fusion-associated genes as localized perturbation sources and learns how their influence propagates along interaction paths toward disease subgraphs, thereby capturing propagation patterns that link related diseases and contribute to their comorbidity. Experiments on a benchmark disease comorbidity dataset show that FuDiCo outperforms state-of-the-art methods, achieving statistically significant improvements. These results shed light on the importance of gene fusion events in understanding disease relationships.

## 1. Introduction

The term comorbidity was introduced in 1970 to describe additional diseases co-occurring with a primary index disease [1]. In patients with chronic diseases, the presence of coexisting diseases before treatment can substantially influence the clinical course, prognosis, and evaluation of treatment outcomes of the index disease [1]. Over the past decades, comorbidity has remained highly prevalent among individuals with chronic diseases. Recent national surveillance data indicate that the prevalence of two or more chronic diseases among U.S. adults increased from 47.3% in 2013 to 51.4% in 2023 [2]. The increasing prevalence of comorbidity underscores the need for computational approaches that can better capture molecular interaction patterns associated with disease comorbidity.

Early studies characterized disease relationships by identifying shared disease-associated genes [3]. However, genetic overlap alone may not fully capture the complexity of disease relationships, as disease phenotypes typically emerge from the interplay of multiple pathobiological processes within complex molecular interaction networks rather than from abnormalities in a single gene [4]. To effectively model these complex molecular relationships, network medicine conceptualizes diseases as modules within the human protein–protein interaction (PPI) network, where nodes represent gene products, and each disease module is defined by its associated genes [4]. Within the disease module paradigm, disease comorbidity is inferred using a network proximity measure based on the separation between disease modules in the PPI network, with smaller separations indicating stronger comorbidity relationships [5].

Inspired by the concept of network proximity, subsequent approaches embed the PPI network into a lower-dimensional space in which disease-associated gene products are encoded as vectors that preserve network geodesic distances [6,7]. Disease comorbidity is then predicted using disease-level embeddings obtained by aggregating the resulting gene-product vectors. Although these embedding-based approaches effectively capture global connectivity patterns in the PPI network, they represent diseases through global proximity relationships among individual gene products and thus overlook the internal topology of fragmented disease modules, where gene products may form multiple connected components within a module. As a result, these approaches may fail to capture patterns relevant to comorbidity encoded in the component-level topology of fragmented disease modules.

More recent graph deep learning approaches attempt to address these limitations by learning disease module representations from the topology of their fragmented subgraphs. For example, SNN-VGA [8] represents disease modules as fragmented subgraphs and learns topology-aware representations of their connected components through random anchor-patch message passing. FDS-CAP [9] extends this approach by introducing a component-level attention mechanism that adaptively weights connected components according to their contribution to the overall disease-subgraph representation. Advancing this line of work, DisSubFormer [10] replaces random anchor-based message passing with fully learnable subgraph-to-subgraph transformer attention over biologically informed anchor patches.

Despite these advances, subgraph-based modeling approaches learn representations from the topological properties of disease modules within the PPI network that depend on currently annotated disease-associated genes. However, these approaches remain limited by incomplete disease–gene annotation [5] and may therefore fail to fully capture network-level molecular interaction patterns associated with disease comorbidity. Consequently, addressing these limitations requires extending beyond currently annotated disease-associated genes to include additional genes implicated through molecular events such as mutations [11], regulatory disruptions [12], and gene fusions [13]. Among these, gene fusion is defined as the formation of a single transcriptional unit through the juxtaposition of sequences from two previously independent genes, producing a chimeric gene or transcript [14]. Gene fusion events may arise through structural genomic rearrangements, including translocations, inversions, deletions, insertions, or other complex chromosomal alterations that create fusion genes. These fusion genes may subsequently be transcribed into fusion transcripts. Alternatively, fusion transcripts can be produced through RNA-level mechanisms, such as transcriptional read-through between neighboring genes or trans- or cis-splicing of pre-mRNAs [15]. When the open reading frame (ORF) is preserved, these fusion transcripts may be translated into chimeric proteins combining functional domains from distinct genes. Functionally, gene fusions can lead to gain- or loss-of-function effects, including constitutive activation of kinase signaling or deregulation of transcriptional programs [16]. Additionally, gene fusions may disrupt interactions with key cellular regulators, thereby perturbing PPI networks [17]. Consistent with these functional effects, gene fusions are strongly implicated in human diseases and are increasingly recognized as clinically significant molecular events with diagnostic, prognostic, and therapeutic relevance across diverse pathological conditions [13,16,18,19].

Motivated by the growing recognition of gene fusions as clinically significant molecular events and their impact on protein interactions, we propose FuDiCo, a computational framework that models disease comorbidity as gene fusion-initiated influence propagation over the PPI network. In network medicine, molecular events can induce perturbations that are not confined to directly involved gene products but may propagate through molecular interaction networks to influence otherwise genetically intact interactors [4]. Accordingly, when fusion events affect certain genes, the resulting perturbations may spread through the PPI network, influencing additional interacting gene products beyond those directly implicated in the initial event. FuDiCo models this process by representing each fusion-associated gene as a localized perturbation source and learning how the resulting influence propagates through PPI paths toward disease subgraphs. If fusion-initiated influence propagates toward multiple disease subgraphs, the resulting propagation patterns may capture network-level molecular relationships associated with disease comorbidity.

**Figure 1:**
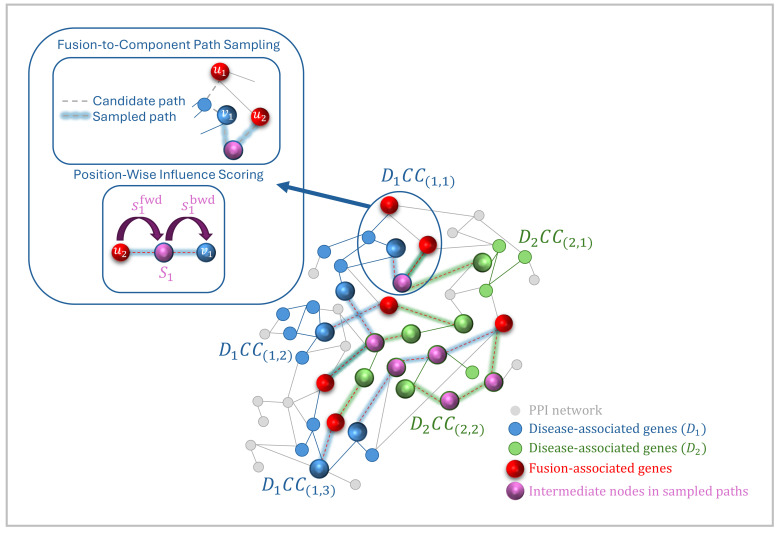
Gene fusion-initiated influence propagation in the protein–protein interaction (PPI) network. The PPI network is modeled as a graph $G_{PPI}$ that serves as the underlying molecular interaction scaffold. Within $G_{PPI}$, genes associated with disease *i* form an induced subgraph $D_{i}$, which is composed of connected components ${CC}_{\left(i,j\right)}$, where *j* is the component index. Fusion-associated genes are represented as localized perturbation source nodes whose influence propagates along interaction paths toward ${CC}_{\left(i,j\right)}$. These propagation paths are subsequently encoded to capture network-level influence patterns that link related diseases and contribute to comorbidity. In the illustrated example of the fusion-to-component path sampling and position-wise influence scoring process, candidate paths connecting fusion-associated genes $u_{1},u_{2}\in {FG}_{1}$ to component node $v_{1}\in {CC}_{\left(1,1\right)}$ form a candidate pool for path sampling. Among these candidates, the path connecting $u_{2}$ to $v_{1}$ is selected and added to $P_{\left(1,1\right)}^{\left(2\right)}$, since $u_{2}$ achieves the highest diffusion-based reachability score to $v_{1}$ (Equation (10)). For the sampled path, the position-wise influence score $S_{1}$ is computed using Equation (13) from the forward and backward scores $\left(s_{1}^{fwd},s_{1}^{bwd}\right)$ to quantify fusion-initiated influence propagation at position t=1 prior to path encoding.

FuDiCo introduces several methodological innovations aimed at extending disease representation learning beyond the topological properties of currently annotated disease genes. Specifically, FuDiCo captures the propagation of fusion-initiated perturbations through sampled propagation paths within the PPI network. These propagation paths are subsequently encoded using a fusion influence-aware gated recurrent unit (GRU) architecture. In this architecture, the recurrent gating mechanisms are conditioned on diffusion-based reachability between fusion-associated genes and disease-subgraph components. Collectively, these methodological innovations form a unified framework for disease comorbidity prediction through fusion-initiated influence propagation. Figure 1 provides a conceptual illustration of this propagation process within the PPI network.

## 2. Materials and Methods

### 2.1. Materials

#### 2.1.1. Protein–Protein Interaction Network

The protein–protein interaction network used in this study consists of 13,460 protein-coding genes connected by 141,296 unique interactions between their encoded protein products [5]. Gene identifiers are mapped to their corresponding protein products using UniProtKB release 2025_03 [20] to represent interactions at the protein level.

#### 2.1.2. Disease–Gene Associations

The disease–gene association dataset used in this study was originally reported in [5]. In that study, disease–gene annotations from Online Mendelian Inheritance in Man [21] and UniProtKB/Swiss-Prot [22] were combined with genome-wide association study data from the Phenotype–Genotype Integrator [23], where only associations meeting a genome-wide significance threshold of $p\leq 5\times 10^{-8}$ were retained. The dataset was further restricted to diseases with at least 20 associated genes and to genes with available interaction data, resulting in a final set of 299 diseases and 3173 unique associated genes.

#### 2.1.3. Relative Risk-Based Comorbid Disease Pairs

Disease-pair relationships are obtained from the analysis of the Medicare dataset reported in [5], which comprises medical records of disease history for 30 million individuals aged 65 years and older. The analysis identified 10,743 disease pairs, each quantified by a relative risk (RR) value that measures co-occurrence beyond what is expected under independence. For a disease pair $\left(D_{i},D_{j}\right)$, the relative risk is defined as

(1) $${RR}_{D_{i}D_{j}}=\frac{C_{D_{i}D_{j}}\cdot N}{P_{D_{i}}\cdot P_{D_{j}}},$$

where $C_{D_{i}D_{j}}$ is the number of patients diagnosed with both diseases, N represents the total population size, and $P_{D_{i}}$ and $P_{D_{j}}$ denote the number of patients diagnosed with the respective diseases. In this study, comorbidity is operationally determined using a predefined threshold θ applied to the RR values, such that a disease pair $\left(D_{i},\;D_{j}\right)$ is classified as comorbid if ${RR}_{D_{i}D_{j}}>\theta$.

#### 2.1.4. Fusion Gene Dataset

The fusion gene dataset is obtained from FusionGDB 2.0 [24]. This resource integrates fusion gene information from two major databases: ChiTaRS 5.0 [25], a database of chimeric transcripts matched with druggable fusions and 3D chromatin maps, and ChimerDB 4.0 [26], an updated and expanded database of curated fusion genes. ChiTaRS contains 50,360 fusion genes, whereas ChimerDB reports 52,737. The union of these two datasets yields 102,647 unique fusion genes in FusionGDB 2.0. ORF annotations are included in FusionGDB 2.0 for fusion events, with categories assigned according to breakpoint location within gene regions of both gene partners, namely coding sequence (CDS), untranslated region, or intron. For fusion events in which both breakpoints occur within coding sequences (CDS–CDS), ORF status is determined from the full-length fusion transcript sequence. If the length of the resulting sequence is a multiple of three, the fusion is classified as in-frame, indicating preservation of the reading frame. Otherwise, it is classified as frame-shift, reflecting disruption of the reading frame. This classification is biologically significant, as preservation or disruption of the reading frame determines whether a continuous coding sequence is maintained in the fusion transcript. When such a continuous coding sequence is preserved, the fusion transcript may be translated into a chimeric protein. Accordingly, we retain ORF annotations for CDS–CDS fusion events, including 16,273 in-frame and 17,803 frame-shift fusions.

### 2.2. Methods

#### 2.2.1. Problem Definition

The PPI network is modeled as an undirected graph $G_{PPI}=\left(V,E\right)$, where $V=\left\{1,\cdots ,n\right\}$ denotes the set of protein-coding genes (nodes), and E⊆V×V represents the undirected edge set, where each edge $\left(u,v\right)\in E$ indicates a protein–protein interaction between the proteins encoded by genes u and v. The graph is equivalently described by an adjacency matrix $A\in {\left\{0,1\right\}}^{n\times n}$, where a matrix element $a_{uv}=1$ if an edge exists between nodes u and v, and $a_{uv}=0$ otherwise.

A node embedding matrix $F\in R^{n\times d}$ contains the initial embedding vectors for all nodes in $G_{PPI}$, where the v-th row corresponds to the embedding vector of node v, denoted by $f_{v}\in R^{d}$. These embedding vectors are derived from a pretrained Evolutionary Scale Modeling (ESM-2) protein language model [27], which generates representations based on the amino acid sequence of the protein encoded by each gene. Each disease is represented by an induced subgraph $D_{i}=\left(V_{i},E_{i}\right)$, where $V_{i}\subset V$ is the set of nodes corresponding to genes associated with the disease, and $E_{i}=\left\{\left(u,v\right)\in E\right|\;u,v\in V_{i}\}$ defines the subset of PPI edges connecting those nodes. Because disease-associated genes may not form a single connected region in $G_{PPI}$, $D_{i}$ may consist of multiple connected components defined as $D_{i}^{\left(CC\right)}=\left\{{CC}_{\left(i,1\right)},\cdots ,{CC}_{\left(i,n_{i}\right)}\right\}$, where $n_{i}$ is the number of connected components. For each component ${CC}_{\left(i,j\right)}$, we compute an initial component representation $h_{{CC}_{\left(i,j\right)}}^{init}\in R^{d_{CC}}$ by aggregating the embedding vectors of its nodes. Given a disease-subgraph set $D=\left\{D_{1},\cdots ,D_{m}\right\}$, FuDiCo learns disease-subgraph representations ${\left\{h_{D_{i}}\right\}}_{i=1}^{m}$ for comorbidity prediction, where $h_{D_{i}}\in R^{d_{D}}$ and m is the number of disease subgraphs.

#### 2.2.2. Model Design Overview

**Figure 2:**
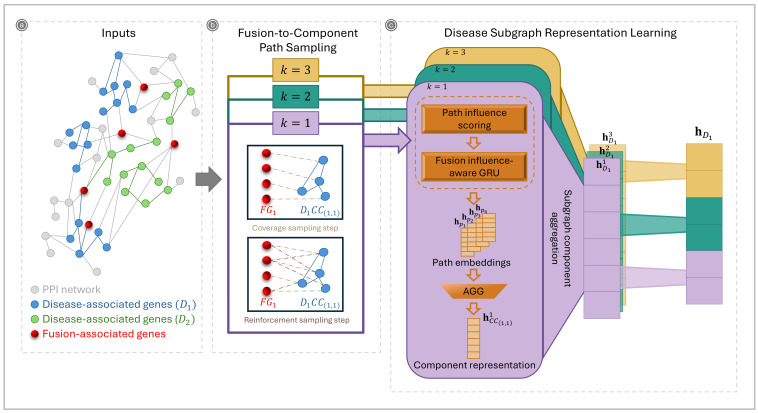
FuDiCo architecture. (**a**) Inputs: the protein–protein interaction (PPI) network, disease-associated genes forming disease subgraphs $D_{i}$ with their connected components ${CC}_{\left(i,j\right)}$, and the corresponding fusion-associated gene sets ${FG}_{i}$; (**b**) fusion-to-component path sampling: for each path length $k\in \left\{1,\;2,\;3\right\}$, paths from fusion-associated genes in ${FG}_{i}$ to nodes within each connected component ${CC}_{\left(i,j\right)}$ of $D_{i}$ are sampled using a two-step sampling procedure; (**c**) disease-subgraph representation learning: sampled paths are scored and encoded using a fusion influence-aware GRU to generate path embeddings $h_{p}$. These path embeddings are aggregated to obtain component representations $h_{{CC}_{\left(i,j\right)}}^{k}$, which are subsequently aggregated into path-length-specific disease-subgraph representations $h_{D_{i}}^{k}$. The resulting representations are then combined to produce the final disease-subgraph representation $h_{D_{i}}$ for comorbidity prediction.

We consider the PPI graph $G_{PPI}$ as the underlying molecular interaction scaffold that supports gene fusion-initiated influence propagation, where each disease is represented as an induced subgraph $D_{i}$ within $G_{PPI}$. On this scaffold, FuDiCo defines genes involved in gene fusion events as sources of perturbation and models how their influence propagates across paths of varying lengths toward disease subgraphs. The resulting propagation patterns are then encoded as disease-subgraph representations (Figure 2). An algorithmic overview of FuDiCo is provided in Algorithm A1 (Appendix A).

#### 2.2.3. Fusion-to-Component Diffusion Reachability

For each disease subgraph $D_{i}$, we first determine the fusion-associated genes that function as sources of fusion-initiated influence propagation. These genes are derived from fusion events that occur either within $D_{i}$, where both partners belong to $V_{i}$, or across its boundary, involving at least one gene in $V_{i}$. Because both cases can introduce disease-relevant influence, we unify the participating fusion-associated genes into a single source set ${FG}_{i}$, which defines the initiating genes for influence propagation toward the disease subgraph $D_{i}$. From this unified source set, influence propagates node-to-node along interaction paths in $G_{PPI}$, reaching nodes in each connected component ${CC}_{\left(i,j\right)}$ of $D_{i}$. Formally, interaction paths are defined by

(2) $$\{\left(v_{0},\cdots ,v_{k}\right):v_{0}\in {FG}_{i},v_{k}\in {CC}_{\left(i,j\right)},(v_{t-1},v_{t})\in E,\;\;for\;t=1,\cdots ,k\},$$

To quantify fusion-initiated influence propagation from fusion-associated genes to nodes in each connected component, we define a symmetric degree-normalized diffusion operator P on $G_{PPI}$ as follows:

(3) $$P={Deg}^{-\frac{1}{2}}A\;{Deg}^{-\frac{1}{2}},$$

where A is the adjacency matrix of $G_{PPI}$, and $Deg\in R^{n\times n}$ is the diagonal degree matrix with entries:

(4) $${Deg}_{uv}=\left\{\begin{array}{c} \deg\left(u\right),\;\;\;u=v\\ 0,\;\;\;\;\;\;\;\;\;\;\;\;\;\;u\neq v \end{array}\right.,\;\;where\;\deg\left(u\right)=\sum_{w=1}^{n}a_{uw},$$

Node-to-node connectivity in $G_{PPI}$ is measured by the number of walks between them, which follows the recursive relation of matrix powers [28]:

(5) $${\left(A^{k+1}\right)}_{uv}={\left(A^{k}A\right)}_{uv}=\sum_{w=1}^{n}{\left(A^{k}\right)}_{uw}a_{wv},$$

where ${\left(A^{k+1}\right)}_{uv}$ gives the number of walks of length k+1 from node u to node v. Since the diffusion operator P is a normalized form of A, ${\left(P^{k}\right)}_{uv}$ captures degree-normalized walks of length k between nodes. However, considering only a single walk length k may fail to capture the full propagation extent, since influence may propagate across walks of varying lengths. Accordingly, we define a truncated diffusion operator to aggregate degree-normalized walks across different lengths as follows:

(6) $$T_{K}=\sum_{k=1}^{K}w_{k}P^{k},$$

where K∈N denotes the maximum walk length and ${\left\{w_{k}\right\}}_{k=1}^{K}$ are normalized length weights given by $w_{k}=\frac{\beta^{k}}{\sum_{i=1}^{K}\beta^{i}}$ for $\beta \in \left(0,1\right)$, such that $\sum_{k=1}^{K}w_{k}=1$. Smaller values of β place proportionally greater weight on shorter walks, thereby emphasizing local influence propagation.

Using the truncated diffusion operator, we characterize the diffusion-based reachability for any node pair $\left(u,v\right)$ as

(7) $$R_{K}\left(u\to v\right)≜{{(T}_{K})}_{uv},$$

For a fusion-associated gene $u\in {FG}_{i}$ and a node $v\in {CC}_{\left(i,j\right)}$, $R_{K}\left(u\to v\right)$ quantifies the strength of fusion-initiated influence that can propagate from u to v through interaction paths of length at most K in $G_{PPI}$. For length-specific analysis, we also use $R_{k}\left(u\to v\right)$ with k≤K, which is computed by limiting the diffusion operator to walks of length up to k.

#### 2.2.4. Fusion-to-Component Path Sampling

For each fusion-associated gene $u\in {FG}_{i}$ and each node $v\in V_{i}$ in the disease subgraph $D_{i}$, we define a pool of candidate simple paths of length $k\in \left\{1,\cdots ,K\right\}$ from u to v in $G_{PPI}$, through which fusion-initiated influence may propagate:

(8) $$C_{i}^{\left(k\right)}\left(u,v\right)=\left\{p=\left(v_{0},\cdots ,v_{k}\right)\;\right|v_{0}=u,\;v_{k}=v,\;\left(v_{t-1},v_{t}\right)\in E,\;\;for\;t=1,\cdots ,k\},$$

For each component ${CC}_{\left(i,j\right)}$ within $D_{i}$ and path length $k\in \left\{1,\cdots ,K\right\}$, FuDiCo samples a set of paths from the candidate pool that (i) ensures coverage across component nodes by selecting at least one path per node, and (ii) allocates the remaining budget to additional paths connecting high-reachability fusion-associated genes to component nodes. Accordingly, a sampled path set for component ${CC}_{\left(i,j\right)}$ is defined from the candidate paths as follows:

(9) $$P_{\left(i,j\right)}^{\left(k\right)}\subseteq \{p\;|\;\exists \;u\in {FG}_{i},\;v\in {CC}_{\left(i,j\right)}:p\in C_{i}^{\left(k\right)}\left(u,v\right)\},|P_{\left(i,j\right)}^{\left(k\right)}|\leq B,$$

where $P_{\left(i,j\right)}^{\left(k\right)}$ denotes the sampled set of paths of length k connecting fusion-associated genes in ${FG}_{i}$ to nodes in the component ${CC}_{\left(i,j\right)}$, and B is a component-level budget that caps the number of sampled paths for each component and path length, ensuring computational tractability through controlled path sampling. Specifically, the sampled path set $P_{\left(i,j\right)}^{\left(k\right)}$ is constructed using a two-step sampling procedure that balances coverage and reinforcement:

• Coverage sampling step: For each component node $v\in {CC}_{\left(i,j\right)}$, we select the fusion-associated gene $u\in {FG}_{i}$ that maximizes diffusion-based reachability (Equation (7)) to v among fusion-associated genes with at least one candidate path of length k ending at v:
  (10) $$u^{⋆}\left(v\right)=\arg\underset{\begin{array}{c} u\in {FG}_{i}\\ C_{i}^{\left(k\right)}\left(u,v\right)\neq \emptyset \end{array}}{max}R_{k}\left(u\to v\right),$$
  We then add at most one path from the selected fusion-associated gene $u^{⋆}\left(v\right)$ ending at v to the sampled path set $P_{\left(i,j\right)}^{\left(k\right)}$.
• Reinforcement sampling step: If ${|P}_{\left(i,j\right)}^{\left(k\right)}|<B$ after the coverage step, we expand the sampled path set $P_{\left(i,j\right)}^{\left(k\right)}$ by allocating the remaining budget to additional paths connecting high-reachability fusion-associated genes to component nodes, while preserving diversity across component nodes.

#### 2.2.5. Fusion-to-Component Path Influence Scoring

For each path $p=\left(v_{0},\cdots ,v_{k}\right)$ in the sampled path set $P_{\left(i,j\right)}^{\left(k\right)}$, where k represents the path length, we quantify fusion-initiated influence propagation at each path position t, with $v_{t}$ corresponding to the associated node. In particular, we assess whether fusion-initiated influence (i) reaches $v_{t}$ within t hops and (ii) can further propagate from $v_{t}$ to the component endpoint node $v_{k}$ within the remaining k−t hops. Therefore, path-level propagation is characterized through position-level influence reception and subsequent influence propagation along the path. This position-level influence is quantified using diffusion-based reachability over $G_{PPI}$ (Equation (7)), through forward and backward scores defined at position t as follows:

(11) $$s_{t}^{fwd}=R_{t}\left(v_{0}\to v_{t}\right),$$

(12) $$s_{t}^{bwd}=R_{k-t}\left(v_{t}\to v_{k}\right),$$

The forward score $s_{t}^{fwd}$ quantifies the strength of influence propagation from the fusion-associated gene source $v_{0}$ to $v_{t}$, while the backward score $s_{t}^{bwd}$ measures the ability of the remaining k−t hops to propagate that influence from $v_{t}$ toward the component endpoint node $v_{k}$. Because the forward and backward scores jointly characterize the extent to which position t receives and propagates fusion-initiated influence along the path, we combine them to define the position-wise influence score $S_{t}$ as

(13) $$S_{t}=\frac{{2s}_{t}^{fwd}s_{t}^{bwd}+\lambda \left(s_{t}^{fwd}+s_{t}^{bwd}\right)}{s_{t}^{fwd}+s_{t}^{bwd}+2\lambda },$$

The central term $\frac{{2s}_{t}^{fwd}s_{t}^{bwd}}{s_{t}^{fwd}+s_{t}^{bwd}}$ corresponds to the harmonic mean between the forward and backward scores, ensuring that strong influence is assigned only when both directions contribute to propagation along the path. However, because the values of $s_{t}^{fwd}$ and $s_{t}^{bwd}$ may vary substantially across positions along the path, this harmonic term can become unstable when one of the scores approaches zero. To stabilize this behavior, we incorporate a scale-adaptive pseudocount λ into $S_{t}$, defined as follows:

(14) $$\lambda =\rho \left(s_{t}^{fwd}+s_{t}^{bwd}\right),\rho =\frac{\tau }{1-2\tau \;},\tau \in \left(0,\;0.5\right),$$

The position-wise influence score $S_{t}$ satisfies several desirable properties including: (i) symmetry, $S_{t}\left(s_{t}^{fwd},s_{t}^{bwd}\right)=S_{t}\left(s_{t}^{bwd},s_{t}^{fwd}\right);$ (ii) boundedness, $\min\left\{s_{t}^{fwd},s_{t}^{bwd}\right\}{\leq S}_{t}\leq \max\left\{s_{t}^{fwd},s_{t}^{bwd}\right\}$; (iii) scale equivariance, such that for any c>0, $S_{t}\left({cs}_{t}^{fwd},{cs}_{t}^{bwd}\right)=cS_{t}$; (iv) a fixed-point property, $S_{t}\left(s,s\right)=s;$ and (v) controlled one-sided behavior, $S_{t}\left(s_{t}^{fwd},0\right)=\tau s_{t}^{fwd},\;S_{t}\left(0,s_{t}^{bwd}\right)=\tau s_{t}^{bwd}$.

#### 2.2.6. Path Encoding with Fusion Influence-Aware GRU

Given the fusion-to-component path set $P_{\left(i,j\right)}^{\left(k\right)}$ with quantified position-wise influence scores along each path, FuDiCo encodes these paths into fixed-length embeddings conditioned on the propagated fusion-initiated influence. For each position $t\in \left\{0,\cdots ,k\right\}$ within the path, we represent the node $v_{t}$ by its embedding $f_{v_{t}}$ and associate it with the position-wise influence score $S_{t}$, which measures the strength of influence propagation at that position. However, propagation strength should capture both the influence at the current position t and the persistence of propagation across preceding positions along the path. Therefore, we introduce an accumulated influence score ${\bar{S}}_{t}$ that aggregates the influence propagation strength across preceding positions up to t as follows:

(15) $${\bar{S}}_{t}=\left(1-\gamma \right)S_{t}+\gamma {\bar{S}}_{t-1},$$

where $\gamma \in \left(0,1\right)$ is a propagation parameter controlling the contribution of previously accumulated influence ${\bar{S}}_{t-1}$ to ${\bar{S}}_{t}$, such that larger γ increases the contribution of past influence, whereas smaller γ places greater emphasis on the current influence score $S_{t}$. The accumulated influence score ${\bar{S}}_{k}$ at the final position k thereby defines a path-wise influence score, capturing the overall strength and consistency of fusion-initiated influence propagated along the path from $v_{0}$ to $v_{k}$.

We encode each path using a fusion influence-aware GRU, built upon the standard GRU architecture [29,30], with gates explicitly conditioned on fusion-initiated influence scores. The fusion influence-aware GRU processes path positions sequentially, maintaining a hidden state that encodes the fusion-initiated influence propagated up to each position. The hidden state at position t, denoted by $h_{t}\in R^{H}$, is computed as a gated combination between the previous state $h_{t-1}\in R^{H}$ and a candidate state ${\tilde{h}}_{t}\in R^{H}$:

(16) $$h_{t}=\left(1-z_{t}\right)⊙h_{t-1}+z_{t}⊙{\tilde{h}}_{t},$$

where ⊙ denotes element-wise multiplication, and $z_{t}\in {\left(0,1\right)}^{H}$ is the update gate computed as

(17) $$z_{t}=\sigma \left(W_{z}f_{v_{t}}+U_{z}h_{t-1}+V_{z}S_{t}\right),$$

where $f_{v_{t}}$ represents the embedding of node $v_{t}$, $W_{z}$, $U_{z}$, and $V_{z}$ are update-gate learnable parameters, and $\sigma \left(\cdot \right)$ is the sigmoid activation function. The update gate is conditioned on the position-wise influence score $S_{t}$, allowing positions with strong propagated influence to exert greater control over the hidden state update. Biologically, path positions corresponding to molecular interactions receiving stronger propagated fusion-initiated influence contribute more strongly to the encoded path representation. As a result, the model prioritizes interaction regions that may be more relevant to disease-associated molecular perturbations underlying disease comorbidity.

Similarly, the reset gate $r_{t}\in {\left(0,1\right)}^{H}$ regulates how much previously propagated fusion-initiated influence is incorporated when forming the candidate state, and is computed as

(18) $$r_{t}=\sigma \left(W_{r}f_{v_{t}}+U_{r}h_{t-1}+V_{r}{\bar{S}}_{t}\right),$$

where $W_{r}$, $U_{r}$, and $V_{r}$ are reset-gate learnable parameters. The reset gate is conditioned on the accumulated influence score ${\bar{S}}_{t}$, modulating the extent to which previously propagated fusion-initiated influence encoded in $h_{t-1}$ contributes to the computation of the candidate state ${\tilde{h}}_{t}$. This design enables the model to preserve persistent fusion-initiated influence propagation patterns across consecutive biologically connected interaction paths.

The candidate state combines the current node embedding, the previous hidden state modulated by the reset gate, and the position-wise influence score $S_{t}$:

(19) $${\tilde{h}}_{t}=\tanh\left(W_{n}f_{v_{t}}+U_{n}\left(r_{t}⊙h_{t-1}\right)+V_{n}S_{t}\right),$$

where $W_{n},U_{n},$ and $V_{n}$ are learnable parameters and $tanh\left(\cdot \right)$ denotes the hyperbolic tangent activation function. Incorporating $S_{t}$ into the candidate state computation enables positions along the path with strong influence propagation to exert a stronger impact on the candidate state representation.

After processing all positions along the path p, the final hidden state $h_{k}$ at position k is taken as the path embedding $h_{p}\in R^{H}$. In addition, the accumulated influence score ${\bar{S}}_{k}$ at the final position k defines the path-wise influence score ${\bar{S}}_{p}$. Together, the pair $\left({h_{p},\bar{S}}_{p}\right)$ is subsequently used to construct disease-subgraph representations for comorbidity prediction. Accordingly, all fusion influence-aware GRU parameters, including those of the update gate, reset gate, and candidate state transformations (i.e., $W_{z},U_{z},V_{z},\;W_{r},U_{r},V_{r},W_{n},U_{n},$ and $V_{n}$), are learned jointly in an end-to-end manner via backpropagation from the final comorbidity prediction loss (Equation (25)). This training procedure thereby learns path representations that capture fusion-initiated influence propagation patterns in the PPI network relevant to disease comorbidity.

#### 2.2.7. Disease-Subgraph Representation and Comorbidity Prediction

**Disease**-**Subgraph Representation.** Given the sampled fusion-to-component paths, the corresponding path embeddings, and the associated path-wise influence scores, we construct disease-subgraph representations that encode fusion-initiated influence propagation across their connected components. For each component ${CC}_{\left(i,j\right)}$ of the disease subgraph $D_{i}$, we use sampled path sets $P_{\left(i,j\right)}^{\left(k\right)}$ corresponding to different path lengths $k\in \left\{1,\cdots ,K\right\}$. Each path $p\in P_{\left(i,j\right)}^{\left(k\right)}$ is associated with a path embedding $h_{p}$ and its corresponding influence score ${\bar{S}}_{p}$. The score ${\bar{S}}_{p}$ is then normalized via a softmax over the influence scores of all paths in $P_{\left(i,j\right)}^{\left(k\right)}$ to yield a path-level attention weight:
  (20) $$\alpha_{p}=\frac{\exp\left({\bar{S}}_{p}\right)}{\sum_{\overset{´}{p}\in P_{\left(i,j\right)}^{\left(k\right)}}\exp\left({\bar{S}}_{\overset{´}{p}}\right)},$$

A weighted representation for component ${CC}_{\left(i,j\right)}$ at path length k is computed as the attention-weighted sum of path embeddings in $P_{\left(i,j\right)}^{\left(k\right)}$, allowing paths with stronger and more persistent fusion-initiated influence propagation to contribute more strongly to the component representation:

(21) $$h_{{CC}_{\left(i,j\right)}}^{k}=\sum_{p\in P_{\left(i,j\right)}^{\left(k\right)}}\alpha_{p}h_{p},$$

The representations of the connected components within $D_{i}$ are further aggregated to obtain a disease-subgraph representation corresponding to path length k:

(22) $$h_{D_{i}}^{k}={AGG}_{CC}\left(h_{{CC}_{\left(i,1\right)}}^{k},\cdots ,h_{{CC}_{\left(i,n_{i}\right)}}^{k}\right),$$

Finally, the overall disease-subgraph representation is constructed by aggregating the path-length-specific disease-subgraph representations across path lengths k:

(23) $$h_{D_{i}}=READOUT\left(h_{D_{i}}^{1},\cdots ,h_{D_{i}}^{K}\right),$$

**Comorbidity Prediction.** For a pair of diseases $D_{i}$ and $D_{j}$, FuDiCo estimates their comorbidity by passing the concatenated disease-subgraph representations through a two-layer multilayer perceptron (MLP) followed by a sigmoid activation:

(24) $$\hat{y}=\sigma \left(W_{2}ReLU\left(W_{1}concat\left(h_{D_{i}},h_{D_{j}}\right)+b_{1}\right)+b_{2}\right),$$

where $\hat{y}\in \left(0,1\right)$ denotes the predicted probability that diseases $D_{i}$ and $D_{j}$ are comorbid, $W_{1}$ and $W_{2}$ are learnable weight matrices, and $b_{1}$ and $b_{2}$ are learnable bias vectors of the MLP. The rectified linear unit (ReLU) is used as the activation function.

**Training Objective.** Let ${\left\{\left(D_{i_{n}},D_{j_{n}},y_{n}\right)\right\}}_{n=1}^{N}$ be the set of disease pairs with comorbidity labels, where N is the total number of pairs in the dataset. For the n-th pair, $y_{n}\in \left\{0,1\right\}$ represents the ground-truth comorbidity label, with $y_{n}=1$ indicating that diseases $D_{i_{n}}$ and $D_{j_{n}}$ are comorbid and $y_{n}=0$ otherwise. Given this set and the predicted comorbidity probabilities, the model is trained by minimizing the binary cross-entropy (BCE) loss:
  (25) $$L_{BCE}=-\frac{1}{N}\sum_{n=1}^{N}\left(y_{n}\log{\hat{y}}_{n}+\left(1-y_{n}\right)\log\left(1-{\hat{y}}_{n}\right)\right),$$

### 2.3. Experimental Setup

#### 2.3.1. Dataset

We trained and evaluated FuDiCo using the data sources described in Section 2.1. These sources include: (i) the PPI network, which serves as the base interaction graph; (ii) disease-associated genes represented as induced disease subgraphs of the PPI network; (iii) clinically reported disease pairs with RR values for deriving ground-truth comorbidity labels; and (iv) fusion gene data for modeling fusion-initiated influence.

Following prior work, we set the RR threshold to θ=0 and classify a disease pair ${(D}_{i},D_{j})$ as comorbid if ${RR}_{D_{i}D_{j}}>\theta$, yielding 8874 comorbid disease pairs out of 10,743 total pairs. The 8874 comorbid disease pairs were partitioned at the disease-pair level into 80% training, 10% validation, and 10% testing splits. Under this setting, disease pairs were partitioned disjointly across splits, while individual diseases may appear in multiple splits through different disease-pair combinations. Nevertheless, FuDiCo does not learn disease representations directly from disease-pair comorbidity relations, but rather through fusion-initiated influence propagation within the PPI network, which reduces the risk of information leakage arising from disease overlap across splits. For training and evaluation, negative disease pairs corresponding to 25% of the number of comorbid disease pairs within each split were sampled with replacement from disease pairs not included in the observed comorbid set. This sampling ratio was selected to preserve class imbalance characteristics commonly observed in real-world clinical and biological association datasets while increasing the proportion of negative samples to make the prediction task more challenging during training and evaluation. In addition, using substantially fewer negative samples relative to positive comorbid pairs could bias model optimization toward the majority positive class and amplify the effect of the highly positive-skewed class distribution on evaluation metrics and reported model performance. Therefore, to ensure fair comparative evaluation under the selected sampling configuration, the same train/validation/test partitions and negative sampling protocol were consistently applied across all the compared methods.

The fusion gene dataset used in FuDiCo is obtained from FusionGDB 2.0, which reports 16,273 in-frame and 17,803 frame-shift fusion events. After combining these two categories and removing duplicates, we obtained 26,956 fusion gene pairs involving 12,751 unique genes. These fusion gene pairs were further filtered to retain only pairs with both partner genes present in the PPI network. The final set comprised 18,360 fusion gene pairs and 9247 genes, covering 68.70% of the genes in the PPI network.

#### 2.3.2. Pretraining Gene Embeddings

Each node in $G_{PPI}$, corresponding to a protein-coding gene, was initialized using protein-sequence embeddings derived from ESM-2. Genes were mapped to UniProtKB protein accessions using the UniProt ID Mapping service, and the associated amino acid sequences for these accessions were retrieved. The sequences were encoded using the 33-layer ESM-2 transformer model (≈650 M parameters) via the Hugging Face Transformers library [31], generating residue-level embeddings that were subsequently mean-pooled to obtain fixed-length sequence representations. When multiple protein sequences corresponded to a single gene, their embeddings were averaged to form a unified gene-level embedding ${f_{v}\in R}^{1280}$. Each gene embedding $f_{v}$, representing the initial node embedding, was then transformed through path-length-specific learnable linear projections from $R^{1280}$ to $R^{128}$. These projections were optimized jointly in an end-to-end manner via backpropagation from the final comorbidity prediction loss (Equation (25)).

#### 2.3.3. FuDiCo Training for Disease Comorbidity Prediction

FuDiCo limits path lengths to $k\in \left\{1,\;2,\;3\right\}$, where K=3 denotes the maximum path length. For each path length k, a component-level path budget of B=100 was applied to control the number of sampled paths. Fusion-initiated influence propagation along these sampled paths was quantified through diffusion-based reachability scores $R_{K}\left(u\to v\right)$ derived from the truncated diffusion operator with normalized length weights $w_{k}$, where β=0.5 (Equation (6)). These reachability scores were subsequently used to compute the position-wise influence scores $S_{t}$, with a smoothing parameter τ=0.2. The accumulated influence score ${\bar{S}}_{t}$ was then obtained using a globally shared propagation parameter γ across all the disease subgraphs, propagation paths, and path lengths. The parameter γ was defined as $\gamma =\sigma \left(\gamma_{raw}\right)$, where $\gamma_{raw}$ is a learnable raw parameter initialized to 0.5. The raw parameter $\gamma_{raw}$ was optimized jointly with all the model parameters via end-to-end backpropagation from the comorbidity prediction objective (Equation (25)).

Initial component representations $h_{{CC}_{\left(i,j\right)}}^{init}\in R^{128}$ were first computed for connected components within the disease subgraph $D_{i}$ via mean aggregation of node embedding vectors within each component. For each path length k, the sampled fusion-to-component propagation paths were encoded using a fusion influence-aware GRU with a hidden-state dimension of 128, yielding path embeddings $h_{p}\in R^{128}$. Within each component, an attention-weighted sum of path embeddings was computed to construct a component-level representation. The resulting component representations were then averaged to obtain a path-length-specific disease-subgraph representation. Disease-subgraph representations corresponding to different path lengths k were subsequently concatenated to form the final disease-subgraph representation $h_{D_{i}}\in R^{32K}$.

FuDiCo was implemented using PyTorch Geometric v2.6.0 [32] within the PyTorch Lightning v2.4.0 training framework [33]. The model was optimized using the AdamW optimizer [34] with weight decay $2.58\times 10^{-4}$. The learning rate was scheduled according to the OneCycleLR policy [35], starting from $8.03\times 10^{-4}$ and increasing to a peak value of $1.41\times 10^{-3}$ during training. Gradient clipping with a threshold of $2.56\times 10^{-1}$ was additionally applied to stabilize optimization. Training was performed for up to 20 epochs with a batch size of 32, and the model with the highest validation area under the receiver operating characteristic curve (AUROC) was selected. All the experiments were conducted on a machine equipped with an NVIDIA GeForce RTX 4070 GPU with 12 GB VRAM, a 24-core Intel Core i9 CPU at 2.00 GHz, and 32 GB RAM.

#### 2.3.4. Comparison on Disease Comorbidity Prediction

We benchmarked FuDiCo against three recent state-of-the-art methods for disease comorbidity prediction: Biologically Supervised Embedding (BSE) [7], FDS-CAP, and DisSubFormer.

BSE derives gene embeddings for all genes in the PPI network by mapping them into a biologically supervised lower-dimensional space while preserving global network structure. Disease-level representations are then obtained by aggregating disease-associated gene embeddings, which are used as input to a supervised support vector machine classifier for comorbidity prediction.

FDS-CAP is a two-stage graph-based deep learning framework that models disease modules as fragmented subgraphs within the PPI network. In the first stage, a subgraph neural network performs topology-aware message passing between connected components and randomly sampled anchor patches, producing component embeddings that are aggregated into disease embeddings using an attention mechanism. In the second stage, these embeddings serve as node features for a variational graph autoencoder applied to an RR-derived human disease network to predict missing comorbidity links.

DisSubFormer further advances subgraph-based disease modeling by introducing a subgraph transformer that learns disease representations from fragmented subgraphs. The model first generates unified gene embeddings by integrating PPI-derived molecular interactions with Gene Ontology-based functional information. It then applies subgraph-to-subgraph attention between biologically informed anchor patches and connected components to learn disease-subgraph representations that capture their topological properties for comorbidity prediction.

## 3. Results

### 3.1. Performance on Disease Comorbidity Prediction

**Figure 3:**
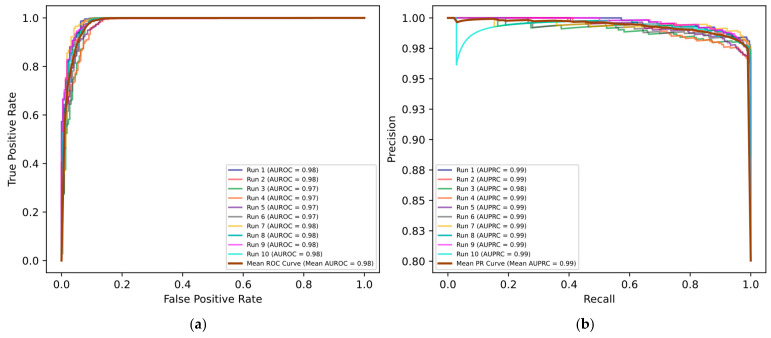
Receiver operating characteristic (ROC) and precision–recall (PR) curves across ten independent runs of FuDiCo: (**a**) ROC curves for individual runs together with the mean ROC curve and the corresponding area under the receiver operating characteristic curve (AUROC) values; (**b**) PR curves for individual runs together with the mean PR curve and the corresponding area under the precision–recall curve (AUPRC) values.

We evaluated FuDiCo for disease comorbidity prediction across ten independent runs using four standard evaluation metrics, including AUROC, accuracy, F1 score, and average precision (AP). As summarized in Table 1, FuDiCo demonstrates consistently strong performance across all the evaluation metrics, achieving mean values of 0.9815 ± 0.0052 for AUROC, 0.9728 ± 0.0046 for accuracy, 0.9828 ± 0.0029 for F1 score, and 0.9940 ± 0.0024 for AP. The relatively small standard deviations observed across these runs indicate that the proposed model is stable and produces reliable predictions. To further examine performance consistency, Figure 3 presents the ROC and precision–recall (PR) curves for individual runs of FuDiCo together with their corresponding mean curves. The curves exhibit minimal variation and substantial overlap, indicating stable performance across independent runs.

**Figure 4:**
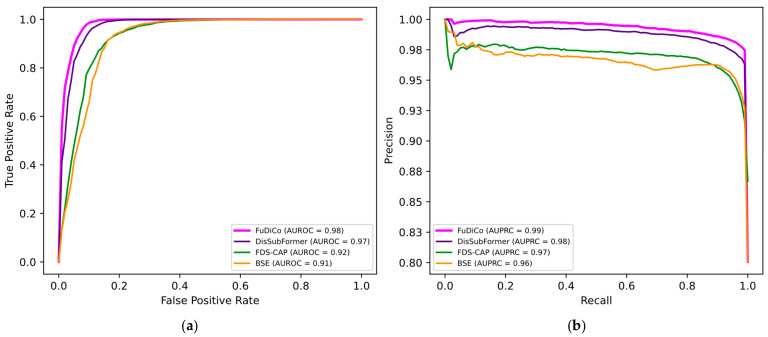
Mean receiver operating characteristic (ROC) and precision–recall (PR) curves across ten independent runs comparing FuDiCo with state-of-the-art models, including DisSubFormer, FDS-CAP, and BSE. (**a**) Mean ROC curves for all models together with the corresponding area under the receiver operating characteristic curve (AUROC) values; (**b**) Mean PR curves for all models together with the corresponding area under the precision–recall curve (AUPRC) values.

We compared FuDiCo with three state-of-the-art methods, namely DisSubFormer, FDS-CAP, and BSE, as shown in Table 1. For all the methods, experiments were repeated over ten independent runs using identical test sets across all the methods to ensure a fair comparison, and the mean performance across runs was reported. Compared to DisSubFormer, FuDiCo improves AUROC by 1.16%, accuracy by 1.26%, F1 score by 0.73%, and AP by 0.54%. Although these performance gains are modest, they remain statistically significant across all the evaluation metrics, with *p*-values of $1.57\times 10^{-4}$ for AUROC, $2.76\times 10^{-5}$ for accuracy, $5.53\times 10^{-5}$ for F1 score, and $3.10\times 10^{-4}$ for AP. These results indicate that the observed improvements are unlikely to arise from random variation across runs. The performance gains become more substantial when comparing FuDiCo with FDS-CAP. FuDiCo achieves statistically significant improvements of 5.68% in AUROC $\left(p=1.59\times 10^{-7}\right)$, 5.41% in accuracy $\left(p=8.55\times 10^{-8}\right)$, 3.19% in F1 score $\left(p=1.26\times 10^{-7}\right)$, and 2.43% in AP $\left(p=9.84\times 10^{-7}\right)$. Relative to BSE, the performance gap further widens, with statistically significant increases of 6.76% in AUROC $\left(p=2.67\times 10^{-7}\right)$, 7.47% in accuracy $\left(p=3.69\times 10^{-12}\right)$, 4.11% in F1 score $\left(p=4.31\times 10^{-12}\right)$, and 2.85% in AP $\left(p=8.40\times 10^{-7}\right)$. Figure 4 further illustrates these comparisons by showing the mean ROC and PR curves across runs for FuDiCo and the state-of-the-art methods. From these curves, FuDiCo maintains higher true positive rates across most false positive rate ranges and higher precision across a broad range of recall values, indicating more reliable identification of comorbid disease pairs. Moreover, the ROC and PR curves of FuDiCo remain consistently above those of the compared methods without noticeable curve crossings, demonstrating stable superiority across different decision thresholds rather than improvements observed only at specific operating points.

The experimental results show that FuDiCo consistently outperforms state-of-the-art methods across all evaluation metrics for disease comorbidity prediction. These performance improvements may be attributed to the explicit incorporation of fusion-initiated influence propagation within the PPI network. By modeling such propagation, FuDiCo learns disease-subgraph representations that better capture disease relationships than approaches relying solely on the topology of known disease–gene associations.

### 3.2. Ablation Study

We conducted an ablation study to evaluate the contribution of the proposed fusion influence-aware GRU to modeling fusion-initiated influence propagation and improving disease comorbidity prediction performance. Specifically, the fusion influence-aware GRU was replaced with a standard GRU architecture that does not incorporate propagation influence scores during sequential propagation path encoding. Under this ablation setting, the fusion influence-aware gating modifications were removed. The position-wise influence score $S_{t}$ was excluded from the update gate and candidate state computations (Equations (17) and (19)), while the accumulated influence score ${\bar{S}}_{t}$ (Equation (18)) was excluded from the reset gate computation. Without these influence-aware modifications, the sampled fusion-to-component propagation paths were encoded using only node embedding sequences derived from the node embedding matrix F. Accordingly, we trained and evaluated FuDiCo with the standard GRU variant while preserving the same propagation path sampling strategy, connected-component aggregation procedure, and overall disease-subgraph representation and comorbidity prediction pipeline used in FuDiCo with the fusion influence-aware GRU.

**Figure 5:**
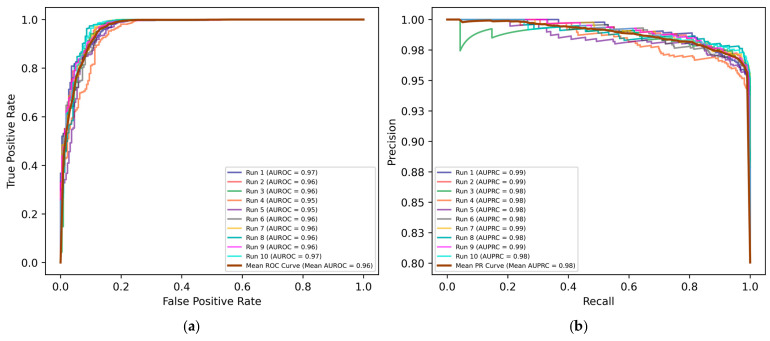
Receiver operating characteristic (ROC) and precision–recall (PR) curves across ten independent runs of FuDiCo with the standard GRU variant: (**a**) ROC curves for individual runs together with the mean ROC curve and the corresponding area under the receiver operating characteristic curve (AUROC) values; (**b**) PR curves for individual runs together with the mean PR curve and the corresponding area under the precision–recall curve (AUPRC) values.

Table 2 summarizes the experimental results comparing FuDiCo with the fusion influence-aware GRU and the standard GRU variant for disease comorbidity prediction across ten independent runs using the standard evaluation metrics. The results demonstrate that removing the fusion influence-aware gating modifications leads to a statistically significant reduction in disease comorbidity prediction performance across all evaluation metrics. Specifically, AUROC, accuracy, F1 score, and AUPRC decreased by 1.67% $\left(p=2.60\times 10^{-6}\right)$, 1.88% $\left(p=3.11\times 10^{-7}\right)$, 1.12% $\left(p=2.56\times 10^{-7}\right)$, and 0.54% $\left(p=1.10\times 10^{-4}\right)$, respectively. To further examine the performance of the standard GRU variant, Figure 5 presents the ROC and PR curves of FuDiCo with the standard GRU variant across ten independent runs together with the corresponding AUROC and AUPRC values.

Beyond the predictive performance, these results further indicate that conditioning the GRU gating mechanism on propagation influence scores enables FuDiCo to prioritize biologically relevant propagation patterns along sampled fusion-to-component paths. Consequently, these prioritized propagation patterns are incorporated into the learned disease-subgraph representations, allowing FuDiCo to better capture comorbid disease relationships associated with fusion-initiated influence propagation.

### 3.3. Scalability and Computational Complexity

The computational complexity of FuDiCo primarily scales with the number of disease subgraphs, connected components, fusion-associated genes, and sampled propagation paths. Accordingly, the overall computational workflow can be divided into two main stages: (1) fusion-to-component path sampling and influence propagation estimation, and (2) sequential propagation path encoding using the fusion influence-aware GRU.

During fusion-to-component path sampling, the number of candidate simple propagation paths may grow substantially with increasing network connectivity and propagation depth, potentially leading to considerable overhead in dense biological interaction networks. To maintain computational tractability, FuDiCo adopts bounded propagation path sampling using fixed path-length and path-budget constraints defined in Section 2.3.3. These constraints limit both the length and number of sampled propagation paths processed during training and inference, thereby controlling memory usage and runtime complexity. In addition, several preprocessing steps, including fusion-to-component path sampling, diffusion-based reachability score computation, and position-wise and accumulated influence score calculation, are performed prior to model training. As a result, this preprocessing accelerates model optimization and hyperparameter tuning.

At the architectural level, FuDiCo models biologically relevant propagation patterns between fusion-associated genes and disease-subgraph connected components rather than modeling exhaustive propagation paths between all nodes in the PPI network and connected components. This design reduces redundant computations by restricting propagation modeling to biologically relevant paths, providing a favorable tradeoff between biological expressiveness and computational efficiency.

In the sequential propagation path encoding stage, the proposed fusion influence-aware GRU scales linearly with both propagation path length and the number of sampled propagation paths, which remains computationally tractable under the bounded path-sampling strategy. To empirically assess runtime scalability, we measured the mean propagation path encoding runtime per training batch across all training epochs, together with the mean encoding runtime associated with each propagation path length k. The mean propagation path encoding runtime was 0.83 s per batch, of which 4%, 23%, and 73% corresponded to propagation path lengths k=1, k=2, and k= 3, respectively. These results indicate that computational cost increases with propagation path length due to the additional sequential operations required for longer fusion-to-component propagation paths, while remaining computationally manageable under the bounded path-sampling strategy used in FuDiCo.

## 4. Discussion

As described in Section 2.3.3, FuDiCo limits propagation path lengths to $k\in \left\{1,\;2,\;3\right\}$ to control the rapid growth in candidate simple paths and reduce computational complexity. To evaluate whether the imposed path length restriction preserves biologically meaningful fusion-initiated influence propagation, we analyzed the distribution of shortest propagation paths between fusion-associated genes and disease-subgraph connected component nodes across all 299 disease subgraphs. Among the 23,302 analyzed nodes in disease-subgraph connected components, all the nodes were reachable from at least one fusion-associated gene within the corresponding disease subgraph through paths in the global PPI network when no path length cutoff is imposed. Under the imposed path length restriction, 99.86% of reachable nodes remained connected to fusion-associated genes within the selected propagation path length cutoff of k≤3, whereas only 0.14% required longer paths.

We further analyzed connected-component-level reachability between disease-subgraph connected components and fusion-associated genes under the imposed propagation path length cutoff. Among the 13,232 analyzed connected components, 13,199 retained at least one node reachable from a fusion-associated gene within k≤3, yielding 99.75% disease-subgraph connected component coverage. The remaining 33 excluded connected components each contain only a single disease-component node requiring propagation paths longer than k=3, indicating that no larger multi-node disease component is entirely excluded by the imposed path length restriction. Accordingly, these findings suggest that biologically meaningful fusion-initiated influence propagation is primarily concentrated within short-range molecular interaction regions of the PPI network. The imposed path length restriction therefore preserves nearly all biologically reachable disease-subgraph connected components while reducing the potential noise associated with long-range propagation patterns in large-scale PPI networks.

Although the reachability analysis shows that nearly all disease-subgraph connected components are reachable within the selected propagation path length cutoff, a small subset of components still lacks valid sampled fusion-to-component paths under the imposed short-range propagation restriction. Such cases may arise from the incomplete coverage of currently available PPI networks and fusion gene datasets, which can limit short-range propagation patterns captured by FuDiCo. For these connected components, FuDiCo falls back to a gated connected-component representation of the form $g^{\left(k\right)}⊙h_{{CC}_{\left(i,j\right)}}^{init,k}$, where $g^{\left(k\right)}=\sigma (\alpha_{k})$ denotes a path-length-specific gate and $\alpha_{k}\in R^{d_{CC}}$ is a learnable parameter vector shared across all connected components at path length k. This gating mechanism controls the extent to which the initial component representation is retained when no valid fusion-to-component paths are available. The gating parameters are optimized jointly with all model parameters via end-to-end backpropagation from the comorbidity prediction objective (Equation (25)). While this gating design preserves model stability and ensures that all components within disease subgraphs are retained, it may slightly reduce the amount of path-specific propagation information available for a small subset of disease subgraphs.

Another limitation of the current study is that FuDiCo does not explicitly distinguish between different biological categories of fusion events, such as germline and somatic events, because such annotations are not available in the utilized fusion gene dataset. However, our method does not assume that fusion events directly drive or causally determine disease comorbidity relationships across all disease contexts. Rather, the proposed framework models how fusion-initiated influence propagation patterns within the PPI network may capture molecular relationships associated with disease comorbidity.

In addition, the dataset is partitioned at the disease-pair level to train and evaluate FuDiCo on the disease comorbidity prediction task, allowing individual diseases to appear in multiple dataset splits through different disease-pair combinations. Although this disease-pair partitioning strategy is commonly adopted in disease relationship prediction studies, disease-level partitioning may provide a more stringent assessment of model generalization to previously unseen diseases. Furthermore, the selected negative sampling strategy may influence the distribution of positive and negative disease pairs used to assess model performance. Future work may investigate the effect of alternative negative sampling configurations on disease comorbidity prediction performance, particularly under different ratios of positive and negative disease pairs and comorbidity label definitions. For example, varying RR thresholds for defining comorbidity labels could alter the balance between positive and negative disease pairs and provide additional perspectives on model robustness.

## 5. Conclusions

In this study, we introduced FuDiCo, a novel framework for disease comorbidity prediction based on gene fusion-initiated influence propagation over the PPI network. Unlike prior methods that rely on the topology or global proximity of annotated disease genes to learn disease representations, FuDiCo integrates gene fusion events, network diffusion, and disease subgraphs to model fusion-initiated influence propagation along sampled paths in the PPI network toward disease subgraphs. The resulting propagation paths are then encoded using a fusion influence-aware GRU encoder to learn disease-subgraph representations that capture the underlying propagation patterns associated with disease comorbidity. Experimental results on a benchmark disease comorbidity dataset demonstrate that FuDiCo achieves robust and consistent state-of-the-art performance for disease comorbidity prediction, outperforming recent methods including DisSubFormer, FDS-CAP, and BSE across multiple evaluation metrics. Beyond predictive performance, these results suggest that gene fusions open new avenues for investigating disease relationships through molecular interaction networks. Future work may further extend this direction by examining how fusion-initiated influence varies across different biological categories of fusion events, including germline and somatic fusion events. Such analyses may reveal category-specific patterns of influence propagation and support future studies of network-based disease comorbidity modeling.

## Figures and Tables

**Table 1:** Performance comparison of FuDiCo and state-of-the-art models for disease comorbidity prediction. Values in bold indicate the best performance for each metric. Reported *p*-values correspond to statistical comparisons between FuDiCo and each model.

Models	Evaluation Metrics							
	AUROC	*p*-Value	Accuracy	*p*-Value	F1	*p*-Value	AP	*p*-Value
FuDiCo (Ours)	**0.9815** ± 0.0052	-	**0.9728** ± 0.0046	-	**0.9828** ± 0.0029	-	**0.9940** ± 0.0024	-
DisSubFormer	0.9703 ± 0.0054	1.57 × 10^−4^	0.9606 ± 0.0056	2.76 × 10^−5^	0.9756 ± 0.0035	5.53 × 10^−5^	0.9886 ± 0.0030	3.10 × 10^−4^
FDS-CAP	0.9288 ± 0.0122	1.59 × 10^−7^	0.9229 ± 0.0095	8.55 × 10^−8^	0.9524 ± 0.0060	1.26 × 10^−7^	0.9704 ± 0.0066	9.84 × 10^−7^
BSE	0.9194 ± 0.0170	2.67 × 10^−7^	0.9052 ± 0.0050	3.69 × 10^−12^	0.9440 ± 0.0028	4.31 × 10^−12^	0.9665 ± 0.0076	8.40 × 10^−7^

**Table 2:** Performance comparison of FuDiCo with the fusion influence-aware GRU and the standard GRU variant for disease comorbidity prediction. Values in bold indicate the best performance for each metric. Reported *p*-values correspond to statistical comparisons between the two FuDiCo variants.

Models	Evaluation Metrics							
	AUROC	*p*-Value	Accuracy	*p*-Value	F1	*p*-Value	AP	*p*-Value
FuDiCo (fusion influence-aware GRU)	**0.9815** ± 0.0052	-	**0.9728** ± 0.0046	-	**0.9828** ± 0.0029	-	**0.9940** ± 0.0024	-
FuDiCo (standard GRU)	0.9654 ± 0.0070	2.60 × 10^−6^	0.9548 ± 0.0049	3.11 × 10^−7^	0.9719 ± 0.0030	2.56 × 10^−7^	0.9887 ± 0.0028	1.10 × 10^−4^

## Data Availability

The raw datasets used in this study are publicly available from previously published sources, including the PPI network, disease–gene associations, and disease-pair datasets reported in [5], as well as the fusion gene dataset from FusionGDB 2.0 [24]. The raw datasets, processed datasets generated in this study, and the FuDiCo source code are publicly available in the following repository: https://github.com/Ashwag-ta/FuDiCo.git, accessed on 3 June 2026.

## Abbreviations

The following abbreviations are used in this manuscript:

AP	Average precision
AUROC	Area under the receiver operating characteristic curve
BCE	Binary cross-entropy
CDS	Coding sequence
ESM-2	Evolutionary Scale Modeling 2
GRU	Gated recurrent unit
MLP	Multilayer perceptron
ORF	Open reading frame
PPI	Protein–protein interaction
PR	Precision–recall
ReLU	Rectified linear unit
ROC	Receiver operating characteristic
RR	Relative risk

## Appendix A

This appendix presents the detailed algorithm of FuDiCo.

**Algorithm A1.** FuDiCo: Gene Fusion-Initiated Path Propagation for Disease Comorbidity Prediction. Forward (fwd) and backward (bwd) scores quantify the strength of fusion-initiated influence propagation along fusion-to-component paths. GRU denotes a fusion influence-aware gated recurrent unit for propagation path encoding. A multilayer perceptron (MLP) is used to predict the probability of comorbidity for a pair of diseases.		
	Input: PPI graph $G_{PPI}=\left(V,E\right)$; Node embeddings {$f_{v}\left|v\in V\right\}$; Disease subgraph set $D=\left\{D_{1},\cdots ,D_{m}\right\}$, where each disease subgraph $D_{i}$ consists of connected components $D_{i}^{\left(CC\right)}=\left\{{CC}_{\left(i,1\right)},\cdots ,{CC}_{\left(i,n_{i}\right)}\right\}$ and fusion-associated gene set ${FG}_{i}$; Maximum path length K; Path budget B; Disease pair set ${\left\{\left(D_{i_{n}},D_{j_{n}}\right)\right\}}_{n=1}^{N}$.	
	Output: Disease subgraph representations ${\left\{h_{D_{i}}\right\}}_{i=1}^{m}$ and predicted comorbidity probabilities ${\left\{{\hat{y}}_{n}\right\}}_{n=1}^{N}$ for disease pairs.	
	Model Parameters: Learnable propagation parameter γ; Parameters of the fusion influence-aware GRU $\theta_{GRU}$; Parameters of the path-length-specific gate $g^{\left(k\right)}$; MLP parameters $W_{1}$, $W_{2}$, $b_{1}$, $b_{2}$.	
	for disease subgraph i=1,⋯,m do	
	for path length k=1,⋯,K do	
	for connected component $j=1,\cdots ,n_{i}$ do	
	$h_{{CC}_{\left(i,j\right)}}^{init,k}={AGG}_{node}\left(\left\{f_{v}|v\in {CC}_{\left(i,j\right)}\right\}\right)$	
	$P_{\left(i,j\right)}^{\left(k\right)}\leftarrow$ SamplePaths $\left({FG}_{i},{CC}_{\left(i,j\right)},k,B\right),\;\;|P_{\left(i,j\right)}^{\left(k\right)}|\leq B$	// See Section 2.2.4
	for each path $p=\left(v_{0},\cdots ,v_{k}\right)\in P_{\left(i,j\right)}^{\left(k\right)}$ do	
	for position t=0,⋯,k do	
	$S_{t}⟵PositionInfluence\left(s_{t}^{fwd},s_{t}^{bwd}\right)$	// See Section 2.2.5
	${\bar{S}}_{t}=\left(1-\gamma \right)S_{t}+\gamma {\bar{S}}_{t-1}$	
	end	
	$h_{p}⟵GRU\left({\left\{f_{v_{t}}\right\}}_{t=0}^{k},{\left\{S_{t}\right\}}_{t=0}^{k},{\left\{{\bar{S}}_{t}\right\}}_{t=0}^{k}\right)$	// See Section 2.2.6
	${\bar{S}}_{p}⟵PathInfluence\left({\bar{S}}_{k}\right)$	
	end	
	${\left\{\alpha_{p}\right\}}_{p\in P_{\left(i,j\right)}^{\left(k\right)}}=softmax\left({\left\{{\bar{S}}_{p}\right\}}_{p\in P_{\left(i,j\right)}^{\left(k\right)}}\right)$	
	$h_{{CC}_{\left(i,j\right)}}^{k}\leftarrow \left\{\begin{array}{cc} \sum_{p\in P_{\left(i,j\right)}^{\left(k\right)}}\alpha_{p}h_{p}, & P_{\left(i,j\right)}^{\left(k\right)}\neq \emptyset \\ g^{\left(k\right)}⊙h_{{CC}_{\left(i,j\right)}}^{init,k}, & \mathrm{otherwise} \end{array}\right.$	
	end	
	$h_{D_{i}}^{k}={AGG}_{CC}\left(h_{{CC}_{\left(i,1\right)}}^{k},\cdots ,h_{{CC}_{\left(i,n_{i}\right)}}^{k}\right)$	// Aggregate subgraph components
	end	
	$h_{D_{i}}=READOUT\left(h_{D_{i}}^{1},\cdots ,h_{D_{i}}^{K}\right)$	// Aggregate across path lengths
	end	
	for disease pair n=1,⋯,N do	
	${\hat{y}}_{n}=\sigma \left(W_{2}ReLU\left(W_{1}concat\left(h_{D_{i_{n}}},h_{D_{j_{n}}}\right)+b_{1}\right)+b_{2}\right)$	// Comorbidity probability
	end	
